# An LFMCW detector with new structure and FRFT based differential distance estimation method

**DOI:** 10.1186/s40064-016-2611-9

**Published:** 2016-06-29

**Authors:** Kai Yue, Xinhong Hao, Ping Li

**Affiliations:** School of Mechatronical Engineering of Beijing Institute of Technology, Beijing, 100081 China

**Keywords:** Collision avoidance radar, Linear frequency modulated continuous wave detector, Intermediate frequency signal based structure, Fractional Fourier transform, Differential distance estimation method

## Abstract

This paper describes a linear frequency modulated continuous wave (LFMCW) detector which is designed for a collision avoidance radar. This detector can estimate distance between the detector and pedestrians or vehicles, thereby it will help to reduce the likelihood of traffic accidents. The detector consists of a transceiver and a signal processor. A novel structure based on the intermediate frequency signal (IFS) is designed for the transceiver which is different from the traditional LFMCW transceiver using the beat frequency signal (BFS) based structure. In the signal processor, a novel fractional Fourier transform (FRFT) based differential distance estimation (DDE) method is used to detect the distance. The new IFS based structure is beneficial for the FRFT based DDE method to reduce the computation complexity, because it does not need the scan of the optimal FRFT order. Low computation complexity ensures the feasibility of practical applications. Simulations are carried out and results demonstrate the efficiency of the detector designed in this paper.

## Background

According to the ‘Global Road Safety Partnership’ site, “Every day, more than 3000 people around the world lose their lives due to road crashes” (Savannah 2011). Many accidents happen as results of driver inattention or bad weather conditions causing poor visibility, as well as pedestrian inattentiveness to their surroundings (Gandhi and Trivedi 2007). So some advanced systems like collision avoidance radars have been designed to control the vehicle in an emergency situation (Lu et al. 2010). In this paper, a LFMCW detector is designed for a collision avoidance radar to estimate the distance between the radar and the vehicle or pedestrian.

Usually, distance between the radar and the vehicle or pedestrian can be obtained by estimating the time delay between the transmit signal and its echo signal. Some common time delay estimation (TDE) have been investigated to detect the distance, which are implemented by finding the peak of cross-correlation between the transmit signal and its echo (Knapp and Carter 1976) or by using adaptive filtering, Hilbert transform and fractional Fourier transform (FRFT) (Grennberg and Sandell 1994; Lin and Chern 1998; Sharma and Joshi 2007; Zhou et al. 2014). Because of the advantage of the FRFT in terms of processing linear frequency modulation (LFM) signals, FRFT are attracting more and more attention in the field of LFM signal processing. However, high computation complexity coming from the scan of the optimal FRFT order restricts its practical applications. Therefore, to utilize FRFT solving FM signal problems in practical applications, a novel intermediate frequency signal (IFS) based structure is designed for the transceiver and a related differential distance estimation (DDE) method based on FRFT is implemented in the signal processor. The LFMCW detector made up of this transceiver and signal processor can estimate the distance effectively.

The rest of this paper is organized as follows. System model is given in “Preliminaries” section. “Proposed LFMCW detector” section describes the proposed IFS based structure and DDE method based on FRFT in detail. In “Simulation and results” section, the simulation results are presented. Finally, the conclusion is drew in the fifth section.

## Preliminaries

**Fig. 1:**
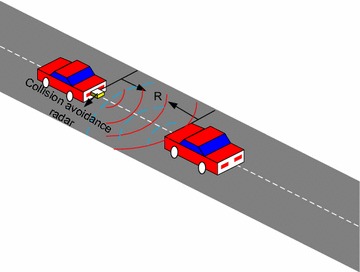
System model

**Fig. 2:**
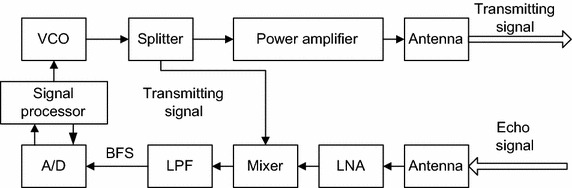
Block diagram of a traditional LFMCW detector

As shown in Fig. 1, a collision avoidance radar can estimate the distance between a vehicle and itself to avoid that the two cars collide. Normally, an FM radar or a pulse radar is used as the collision avoidance radar. Figure 2 describes an FM radar traditional LFMCW detector with the traditional beat frequency signal (BFS) based structure. Usually, correlation, FFT or other methods in time or frequency domain are utilized in this detector to estimate the distance. However, the distance estimation accuracy of these methods is inversely proportional to the modulation band of the FM signal. It means that better distance estimation accuracy needs wider modulation band of the FM signal. Nevertheless, wider modulation band will bring other problems, such as frequency modulation nonlinear, which can affect the distance estimation performance. Therefore we hope a distance estimation method whose accuracy has nothing to do with the modulation band. Then a novel distance estimation method using FRFT is proposed in this paper. However, if we using the method in the LFMCW detector shown as Fig. 2, we have to search for the optimal rotation angle and this makes the computation complexity enhanced obviously.

**Fig. 3:**
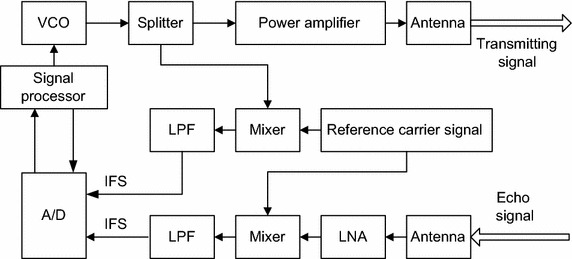
Block diagram of the LFMCW detector proposed in this paper

For avoiding the search for the optimal rotation angle, the LFMCW detector with new IFS based structure is proposed in paper and it is shown in Fig. 3. Compared with the traditional one, the decrease of computation complexity is based on the additional hardware cost. The additional hardware consists of a LPF and a mixer. Either the structure burden or the financial burden of the additional hardware can be ignored under the current level of technology. However, the decrease of computation complexity is multiple, because each search needs carrying out once FRFT.

### Fractional Fourier transform

As a generalization of conventional Fourier transform (FT), the FRFT has been shown to be more effective than the FT in many signal processing areas. Several signal processing methods based on FRFT have been proposed and their properties also have been derived and discussed, such as convolution, filtering, correlation and so on (Ozaktas and Barshan 1994; Almeida 1994; Ozaktas et al. 2001). To make the FRFT come true, some fast discrete fractional Fourier transform (DFRFT) have been done (Ozaktas et al. 1996; Pei and Yeh 1999; Candan and Kutay 2000; Ran et al. 2001). Among them, the Ozaktas’ DFRFT fast algorithm is widely regarded as an efficient one (Ozaktas et al. 1996), which has high precision, fast speed and the computation complexity matched with FFT.

The FRFT with a rotation angle *α* of signal *f*(*t*), denoted as *F*_*α*_(*u*), is defined as (Ozaktas et al. 2001):

1 $$\begin{aligned} F_{\alpha } (u) &= \,\int_{ - \infty }^{ + \infty } {f(t)K_{\alpha } (u,\,t)dt} \hfill \\ f(t) &= \,\int_{ - \infty }^{ + \infty } {F_{\alpha } (u)K_{\alpha }^{ * } (u,\,t)du} \hfill \\ \end{aligned} ,$$

where the transform kernel function *K*_*α*_(*u*, *t*) is given as:

2 $$K_{\alpha } (u,\,t)\, = \,\sqrt {\frac{1 - j\cot \alpha }{2\pi }} \exp \left( {j\frac{{t^{2} + u^{2} }}{2}\cot \alpha - jtu\csc \alpha } \right) ,$$

in which *t* is time, and *u* is the coordinate in fractional Fourier domain (FRFD). Meanwhile *j* is the imaginary unit and the function exp() is the exponential function.

**Fig. 4:**
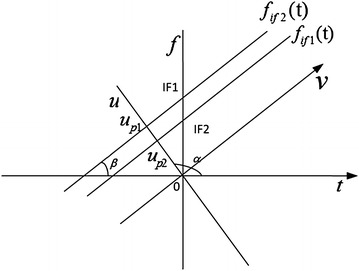
Definition of FRFT in the time–frequency domain

The parameter *α* is the rotation angle of FRFT. When *α* increases from 0 to *π*/2, the FRFT transforms a continuous signal from its time domain to the Fourier image. If *α* or *α* + *π* is multiples of 2*π*, the kernel *K*_*α*_(*u*, *t*) is simplified as *δ*(*t* − *u*) or *δ*(*t* + *u*), respectively. FRFT with rotation angle *α* defined in the time–frequency domain is shown in Fig. 4. In this figure, *t* is time and *f* is frequency. Meanwhile *u* is the coordinate in FRFD and *v* is the amplitude in FRFD. *f*_*if*1_(*t*) and *f*_*if*2_(*t*) are instantaneous frequency of two signals with *IF*1 and *IF*2 as their initial frequency (IF). *u*_*p*1_ and *u*_*p*2_ are peak positions of the two signals in FRFD after FRFT. It is noticed that with the change of the rotation angle *α*, the axis *u* will perpendicular to the instantaneous frequency *f*_*if*1_(*t*) and *f*_*if*2_(*t*) when the rotation angle *α* = *β* + *π*/2. Under this condition, the relationship between the peak positions in FRFD and the initial frequency of the signal can be expressed as

3 $$\begin{aligned} IF1 &= u_{p1} \csc \alpha \\ IF2 &= u_{p2} \csc \alpha \end{aligned}$$

Ozaktas’ DFRFT can be expressed as Ozaktas’ et al. (1996)

4 $$F_{\alpha } \left( {\frac{m}{2\Delta x}} \right)\, = \,\frac{{A_{\alpha } }}{2\Delta x}\sum\limits_{n\, = \, - N}^{N} {\exp \left( {j\pi \gamma \left( {\frac{m}{2\Delta x}} \right)^{2} \, - \,j2\pi \csc \alpha \frac{mn}{{(2\Delta x)^{2} }}\, + \,j\pi \gamma \left( {\frac{n}{2\Delta x}} \right)^{2} } \right)} \,f\left( {\frac{n}{2\Delta x}} \right),$$

where $A_{\alpha } \, = \,\frac{{\exp \left( {{{ - j\pi \text{sgn} (\sin \alpha )} \mathord{\left/ {\vphantom {{ - j\pi \text{sgn} (\sin \alpha )} {4\, + \,{{j\alpha } \mathord{\left/ {\vphantom {{j\alpha } 2}} \right. \kern-0pt} 2}}}} \right. \kern-0pt} {4\, + \,{{j\alpha } \mathord{\left/ {\vphantom {{j\alpha } 2}} \right. \kern-0pt} 2}}}} \right)}}{{\left| {\sin \alpha } \right|^{1/2} }}$ and *γ* = cot *α*·$\frac{1}{2\Delta x}$ is the normalized sampling interval in both time domain and FRFD. This DFRFT can be realized by three steps:

1. Multiply a chirp signal in the time domain;
2. Carry out FFT to the signal after first step.
3. Multiply another chirp signal and we get the DFRFT of the original signal.

We defined that multiplication times of an algorithm is the computation complexity. As a result, the computation complexity of this DFRFT with *N* points are 2*N* + *N*log_2_*N*/2, which is not so more than FFT.

## Proposed LFMCW detector

According to Figs. 2 and 3, it can be noticed that the structure of this detector is different from the traditional one. Traditional LFMCW detectors use the beat frequency signal (BFS) to gain the wanted information, whereas in the proposed detector information is acquired from the IFS. The mixer puts out BFS by mixing the echo signal with the transmit signal. However, IFS is obtained by mixing the echo signal with the reference carrier signal. Compared to BFS, the frequency modulation slope of the transmit signal is remained in the IFS. Because of the knowledge of the frequency modulation slope, FRFT can be carried out without hunting for the optimal order. As a result, the computation complexity of the FRFT based DDE method is significantly low.

Supposed that the LFM transmitting signal *s*_1_(*t*) with amplitude *a*_1_, carrier frequency *f*_1_ and the frequency modulation slope *K*_1_ is expressed as

5 $$s_{1} (t)\, = \,a_{1} \cos 2\pi \left( {f_{1} t\, + \,\frac{1}{2}K_{1} t^{2} } \right) .$$

According to Fig. 2, this signal is mixed with an echo signal *s*_2_(*t*) which can be expressed as

6 $$s_{2} (t)\, = \,a_{1} \lambda_{1} \cos 2\pi \left( {f_{1} \left( {t\,{ - }\,\tau (t)} \right)\, + \,\frac{1}{2}K_{1} \left( {t - \tau (t)} \right)^{2} } \right) ,$$

where *τ*(*t*) is the time delay and *λ*_1_ is the amplitude factor. Then we get the BFS *s*_*bf*_(*t*) and it can be formulated as

7 $$s_{bf} (t)\, = \,a_{bf} \cos 2\pi \left( {f_{1} \tau (t) - \frac{1}{2}K_{1} \tau^{2} (t) + K_{1} t\tau (t)} \right) .$$

Let $\tau (t) = \frac{{2(R_{0} - v_{0} t)}}{c}$ (*R*_0_ is the initial distance and *v*_0_ is the relative speed. *c* is the velocity of electromagnetic wave) and the *s*_*bf*_(*t*) can be expressed as

8 $$s_{bf} (t) = a_{bf} \cos 2\pi \left( {\frac{{4K_{1} R_{0} v_{0} + 2K_{1} R_{0} - 2f_{1} v_{0} }}{c}t - \frac{{2K_{1} v_{0} c + v_{0}^{2} }}{{c^{2} }}t^{2} + \frac{{2f_{1} R_{0} c - 2K_{1} R_{0}^{2} }}{{c^{2} }}} \right) ,$$

where *a*_*bf*_ is the amplitude. We can find that the frequency modulation slope of *s*_*bf*_(*t*) is $\frac{{4K_{1} v_{0} c + 2v_{0}^{2} }}{{c^{2} }}$ and it is unknown. Consequently, we have to make search for the optimal rotation angle to determine it.

On the contrary, according to Fig. 3, the echo signal is mixed with a reference carrier signal *s*_0_(*t*) = cos2*π*(*f*_1_*t*), and we obtain the IFS *s*_*if*_(*t*) which can be expressed as

9 $$s_{if} (t)\, = \,a_{if} \cos 2\pi \left( {f_{1} \tau (t) - \frac{1}{2}K_{1} \tau^{2} (t) - \frac{1}{2}K_{1} t^{2} + K_{1} t\tau (t)} \right) ,$$

where *a*_*if*_ is the amplitude. Considering $\tau (t) = \frac{{2(R_{0} - v_{0} t)}}{c}$, the equation can be rewritten as

10 $$s_{if} (t)\, = \,a_{if} \cos 2\pi \left( {\frac{{4K_{1} R_{0} v_{0} + 2K_{1} R_{0} - 2f_{1} v_{0} }}{c}t - \frac{{K_{1} + 4K_{1} v_{0} c + v_{0}^{2} }}{{2c^{2} }}t^{2} + \frac{{2f_{1} R_{0} c - 2K_{1} R_{0}^{2} }}{{c^{2} }}} \right)$$

Because of the relationship *v*_0_ ≪ *c* in practice, the frequency modulation slope $\frac{{K_{1} c^{2} + 4K_{1} v_{0} c + v_{0}^{2} }}{{c^{2} }}$ of *s*_*if*_(*t*) is approximate *K*_1_. Therefore, the frequency modulation slope of *s*_*if*_(*t*) is known for us and the FRFT with the optimal rotation angle can be carried out without scan for the optimal rotation angle.

Additionally, when the relative speed is small enough, the time delay *τ*(*t*) can be regarded as a constant *τ* for each computation. So the equation can also be formulated as

11 $$s_{if} (t) = a_{if} \cos 2\pi \left( {f_{1} \tau - \frac{1}{2}K_{1} \tau^{2} - \frac{1}{2}K_{1} t^{2} + K_{1} t\tau } \right) .$$

Normally, this equation is utilized for distance estimation. From this equation, we can also find that the frequency modulation slope of *s*_*if*_(*t*) is known for us and the FRFT with the optimal rotation angle can be carried out without scan for the optimal rotation angle.

### Derivation of the DDE method

**Fig. 5:**
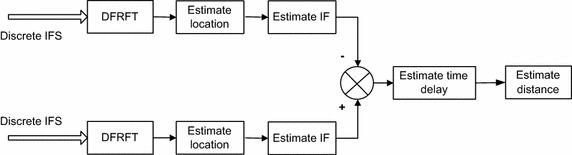
Principle block diagram of the FRFT-based TDE method

The principle block diagram of the FRFT based DDE method is shown in Fig. 5. Two IFSs are sampled by A/D and become discrete signals. Then DFRFT is carried out to them and locations of the two IFSs in fractional Fourier domain are estimated. By means of the locations, IFs of the two IFSs are obtained. Time delay is calculated through the difference of the two IFSs’ IFs. Finally, distance is estimated according to the time delay.

**Fig. 6:**
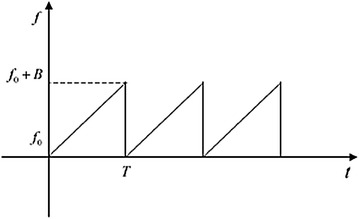
Instantaneous frequency of the saw tooth wave

Supposed that the modulating signal of the LFM signal is a saw tooth wave, then the instantaneous frequency *f*_*if*_(*t*) as shown in Fig. 6 can be denoted as:

12 $$f_{if} (t)\, = \,f_{0} + K(t - nT), \quad nT \le t < (n + 1)T, \quad n = 0,\,1,\,2 \ldots ,$$

where *f*_0_ is the carrier frequency and *T* is the period of the modulated signal. *K* = *B*/*T* is the frequency modulation slope and *B* is the modulation bandwidth. Parameter *n* is the cycle times. So the transmit signal within a modulation period can be expressed as:

13 $$s_{t} (t) = a\cos 2\pi \left[ {\left( {f_{0} - nKT} \right)t + \frac{1}{2}Kt^{2} + \varPhi_{0}^{n} } \right] ,$$

where $\varPhi_{0}^{n}$ = *n*(*n* + 1)*KT*^2^/2 + Φ_0_ (Φ_0_ is the initial phase), and *a* is the amplitude of the transmit signal.

Then, the reflected echo signal can be described as:

14 $$\begin{aligned} s_{r} (t) &= \,\lambda a\cos 2\pi \left[ {(f_{0} - nKT)(t - \tau ) + \frac{1}{2}K(t - \tau )^{2} + \frac{n(n + 1)}{2}KT^{2} + \varPhi_{1} } \right] \\ &= \,\lambda a\cos 2\pi \left[ {(f_{0} - nKT)t - Kt\tau + \frac{1}{2}Kt^{2} + \varPhi_{\tau } } \right], \\ \end{aligned}$$

where Φ_*τ*_ = (*Kτ*^2^ − 2*f*_0_*τ* − 2*nKTτ*)/2 + *n*(*n* + 1)*KT*^2^/2 + Φ_1_, *λ* is the amplitude factor of the echo signal, and the parameter *τ* is the time delay.

The transmit signal and the echo signal are mixed with the reference carrier signal *x*(*t*) = cos 2*πf*_0_*t* in two separate mixers respectively, then the IFSs can be derived as:

15 $$\begin{aligned} s_{it} (t) &= \,\frac{a}{2}\cos 2\pi \left[ { - nKTt + \frac{1}{2}Kt^{2} + \varPhi_{0}^{n} } \right] \hfill \\ s_{ir} (t) & = \,\frac{\lambda a}{2}\cos 2\pi \left[ { - (nKT + K\tau )t + \frac{1}{2}Kt^{2} + \varPhi_{\tau } } \right].\end{aligned}$$

It’s noticed that the frequency modulation slope *K* is remained in the IFSs mentioned as above. So FRFT will be carried out to the IFSs with angle *α* = arctan(*K*) + *π*/2, without hunting for the optimal rotation angle.

Based on the Eq. (15), the IFs *f*_*it*_ and *f*_*ir*_ of the IFSs can be simply obtained as the Eq. (16).

16 $$\begin{aligned} f_{it} &= - nKT \\ f_{ir} &= - (nKT + K\tau ).\end{aligned}$$

Obviously, the time delay *τ* can be estimated through the difference between the *f*_*it*_ and *f*_*ir*_.

According to Eq. (3), while the rotation angle (*α* = arctan(*K*) + *π*/2) is optimal, the relationship between *f*_*ir*_(*f*_*it*_) and the peak position *u*_*pr*_(*u*_*pt*_) in FRFD of the IFSs can be written as

17 $$\begin{aligned} f_{ir} &= u_{pr} \csc \alpha \\ f_{it} & = u_{pt} \csc \alpha.\end{aligned}$$

With the IFs obtained through the estimation of *u*_*pr*_(*u*_*pt*_), the time delay estimation $\widetilde{\tau }$ is achieved and expressed as:

18 $$\widetilde{\tau } = \frac{{u_{pr} - u_{pt} }}{K\sin \alpha }.$$

According to the Eq. (18) and *τ* = 2*R*/*c* (*R* is the distance and *c* is the velocity of electromagnetic wave), the distance estimation value can be formulated as:

19 $$R = \frac{{c\left( {u_{pr} \; - \;u_{pt} } \right)}}{2K\sin \alpha }.$$

### Distance estimation precision and computation complexity

According to Eq. (19), the distance estimation precision of this method is proportional to the estimation accuracy of *u*_*pr*_(*u*_*pt*_). The estimation accuracy of *u*_*pr*_(*u*_*pt*_) is equal to the sampling interval in the fractional domain, and can be expressed as $\Delta u\; = \;{1 \mathord{\left/ {\vphantom {1 {\sqrt N }}} \right.} {\sqrt N }}$ after normalization (Ozaktas et al. 1996). According to the Ozaktas et al. (1996), the real sampling interval Δ*u*_*r*_ can be expressed as

20 $$\Delta u_{r} = \Delta u\sqrt {{{f_{s} } \mathord{\left/ {\vphantom {{f_{s} } T}} \right.} T}} = \frac{{f_{s} }}{N} .$$

where *f*_*s*_ is the sampling frequency in time domain and *N* is the computation point for each processing. Therefore, the distance estimation precision Δ*R* is

21 $$\Delta R = \frac{{cf_{s} \csc \alpha }}{2KN} .$$

For engineering applications, *f*_*s*_ = 10*B* is reasonable. Meanwhile, the frequency modulation slope *K* = *B*/*T*. Consequently, the distance estimation precision can be rewrite as

22 $$\Delta R = \frac{5cT\csc \alpha }{N}$$

**Fig. 7:**
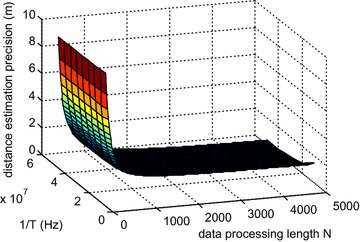
Relationships between the distance estimation precision and the period of the modulated signal or the sampling points

Because parameter *c* and *α* are constants, the period of the modulated signal *T* and sampling points are the main influencing factors for the distance estimation precision. Improvement of the distance estimation precision depends on *T* and *N*, but not the modulation bandwidth. It is beneficial for engineering applications. Figure 7 shows the relationships between the distance estimation precision and the period of the modulated signal or the sampling points.

For the proposed DDE method in this paper, the multiplication times is used to measure its computation complexity. According to Fig. 4, the multiplication times mainly come from the DFRFT of the IFS. As a result, according to the computation complexity of DFRFT, the computation of this DDE method can be expressed as

23 $$2N + {{N\log_{2} N} \mathord{\left/ {\vphantom {{N\log_{2} N} 2}} \right.} 2} .$$

On the contrary, if searching the adaptive angle of DFRFT is needed, the computation complexity with *M* times scan can be written as

24 $$M(2N + {{N\log_{2} N} \mathord{\left/ {\vphantom {{N\log_{2} N} 2}} \right.} 2}) .$$

Compared with Eq. (24), the computation complexity of the proposed DDE method described as Eq. (23) decreases obviously.

## Simulation and results

In this section, simulations are carried out to validate the performance of the detector proposed in this paper. The carrier frequency is 3.0 GHz, and the modulation bandwidth is 30 MHz. The modulation period is 3.3 × 10^−6^ s.

**Fig. 8:**
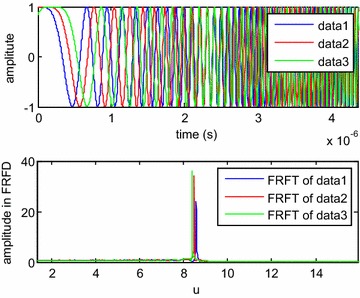
Simulation results of the transmitting signal and echo signals (*above*) and their FRFT (*below*) within a modulation period while the target is stationary relative to the detector

**Fig. 9:**
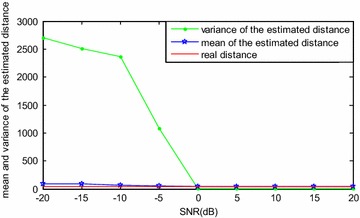
Simulation results of 1000 Monte Carlo simulations with different SNR values

When the target is stationary relative to the LFMCW detector, the time delay between the transmitting signal and the echo signal is a constant. Simulations were carried out for this scenario, and the results are shown in Fig. 8, where data1, data2 and data3 is the transmitting signal, echo signal with time delay of 1e−7 s and echo signal with time delay of 2e−7 s respectively. It can be seen that the locations in the fractional Fourier domain are different for the three signals with different initial frequencies. Moreover, to test the performance of the proposed DDE method with noise, a Monte Carlo simulation with 1000 points was also conducted. The results are shown in Fig. 9. The simulation results prove that the proposed method in this paper is feasible for a fixed-distance measurement when the signal-to-noise ratio (SNR) is no less than 0 dB. This method would certainly be feasible with lower SNR values if the noise was filtered out prior to applying the DFRFT.

**Fig. 10:**
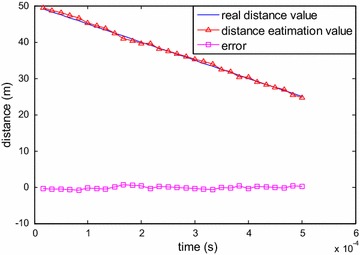
Simulation results of the distance estimation with SNR value 0 dB

If there is some relative velocity between the target and the LFMCW detector, the time delay value will vary. For example, when the target is gradually approaching the system, the time delay will gradually reduce. Simulations of this scenario were carried out, and the results of the instantaneous time delay estimation with SNR value 0 dB are shown in Fig. 10. This figure proves that the proposed DDE method in this paper is also feasible for the instantaneous distance measurements for a moving target.

## Conclusion

In this paper, a novel FRFT based LFMCW detector is proposed to achieve distance measurement for a collision avoidance radar. On the one hand, an FRFT based DDE method is proposed for the detector to realize distance estimation. On the other hand, a new IFS based structure transceiver is designed for the detector to reduce computation complexity of FRFT by taking advantage of the knowledge of frequency modulation slope. Benefited from the FRFT based DDE method and structure, the detector can estimate the distance effectively and make the FRFT feasibly applied in practice rather than merely researched theoretically. Besides, practical FRFT applications in LFM systems have important significance for improving the performance of LFM systems.
